# Profiling lipid mediators in serum from children with H1N1 influenza

**DOI:** 10.1038/s41598-024-66190-y

**Published:** 2024-07-02

**Authors:** Weijun Chen, Yitao Gu, Yongjun Ma, Lele Dong, Liangxuan Pan, Chai Ji, Lanlan Guo, Lianxin Qi, Yuanyuan Zhang, Fei Gao

**Affiliations:** 1 Department of Child Health Care, Children’s Hospital, Zhejiang University School of Medicine, National Clinical Research Center for Child Health, Hangzhou, China; 2 Department of Pediatrics, Shaoxing Shangyu Maternal and Child Health Care Hospital, Shangyu District, Shaoxing, China; 3 Durbrain Medical Laboratory, Hangzhou, 310000 Zhejiang China; 4 Department of Clinical Laboratory, Shaoxing Shangyu Maternal and Child Health Care Hospital, Shangyu District, Shaoxing, China; 5 Department of Pulmonology, Children’s Hospital, Zhejiang University School of Medicine, National Clinical Research Center for Child Health, Hangzhou, China

**Keywords:** Infectious diseases, Biochemistry, Systems biology, Medical research

## Abstract

Influenza A virus subtype H1N1 can cause severe acute respiratory distress syndrome and death in young children and elderly individuals. H1N1 initiates inflammatory responses that aim to contain and eliminate microbial invaders. Various lipid mediators (LMs) are biosynthesized and play a critical role in fighting viruses during inflammation; thus, by profiling the LMs in patients, researchers can obtain mechanistic insights into diseases, such as the pathways disrupted. To date, the relationship between molecular alterations in LMs and the pathogenesis of H1N1 influenza in children is poorly understood. Here, we employed a targeted liquid chromatography coupled with tandem mass spectrometry (LC‒MS/MS) to profile LMs in serum from children with H1N1 influenza (H1N1 children) and recovered children. We found that 22 LM species were altered in H1N1 children with mild symptoms. Analysis of the LM profiles of recovered children revealed a decrease in the levels of thromboxane B2 (TxB_2_) and thromboxane B3 (TxB_3_) and an increase in the levels of other 8 altered LM species associated with H1N1 influenza, including cytochrome P450 (CYP) enzyme-derived dihydroxyeicosatrienoic acids (DiHETrEs) and hydroxyeicosatetraenoic acids (HETEs) from arachidonic acid (AA), and epoxyoctadecamonoenoic acids (EpOMEs) from linoleic acid (LA). Taken together, the results of this study revealed that serum LMs change dynamically in H1N1 children with mild symptoms. The dramatically altered LMs in H1N1 children could serve as a basis for potential therapeutics or adjuvants against H1N1 influenza.

## Introduction

Infection caused by influenza (flu) virus A subtype H1N1 is a major health burden that can be life-threatening, particularly among elderly individuals and patients with comorbid diseases^1^. H1N1 influenza virus can cause significant morbidity and mortality in children^2^. Individuals with the highest rates of influenza-related hospital admission include infants, young children, and adults over 65 years^3^.

While most H1N1 influenza virus infections in humans are self-limiting, highly virulent strains can cause an overexuberant inflammatory response that is detrimental to the host^4^. Although it is well known that the pathogenicity of influenza strains can vary widely, the precise mechanisms leading to the infection outcome are not fully understood. During H1N1 influenza virus-induced inflammation, various lipid mediators (LMs) play important roles in the regulation of inflammatory responses^5,6^. LMs are autacoids produced from essential fatty acids and constitute a central part of the concerted immune response. They are involved in all aspects of the inflammatory response including initiation, propagation, and resolution^7,8^. Although the resolution of inflammation was previously thought to be a passive process, it has since been established that specific LMs play central roles in driving the endogenous counterregulation of inflammation and the activation of resolution^6^.

Arachidonic acid (AA 20:4 n-6), a polyunsaturated fatty acid (PUFA), is the precursor of proinflammatory prostaglandins (PGs), leukotrienes (LTs), thromboxanes (Tx) and proresolving lipoxins (LXs)^9^. The n-3 essential fatty acids eicosapentaenoic acid (EPA 20:5 n-3) and docosahexaenoic acid (DHA 22:5 n-3) are precursors of specialized proresolving mediators (SPMs), namely protectins (PDs), resolvins, and maresins^10^. These molecules play a pivotal role in regulating viral replication as well as in reprogramming the host innate and adaptive immune response^11,12^.

The role of LMs in infection and inflammation has been studied in animal models of viral infection including influenza viruses, herpes simplex viruses, and coronaviruses^6,12^, among which SPMs have been shown to reduce disease severity^11,13^. Since LMs play a critical role in viral infection and inflammation, researchers can uncover disrupted pathways and reveal biological insight into diseases by profiling LMs in patients. The plasma profiling of LMs in coronavirus (COVID-19) patients revealed that the levels of LMs in critically ill patients were reduced, and certain SPMs can be used to determine disease severity^14^. Lu et al.^15^ demonstrated that various eicosanoids were dysregulated in adult patients with H1N1 influenza-induced pneumonia.

In this study, we used a liquid chromatography coupled with tandem mass spectrometry (LC‒MS/MS) platform to characterize the serum LM profiles of healthy children, children with H1N1 influenza (H1N1 children) and children after recovery; in addition, we examined bioactive lipids that were potentially disturbed and related pathways during H1N1 influenza in children.

## Results

### Study design and blood samples

**Figure 1:**
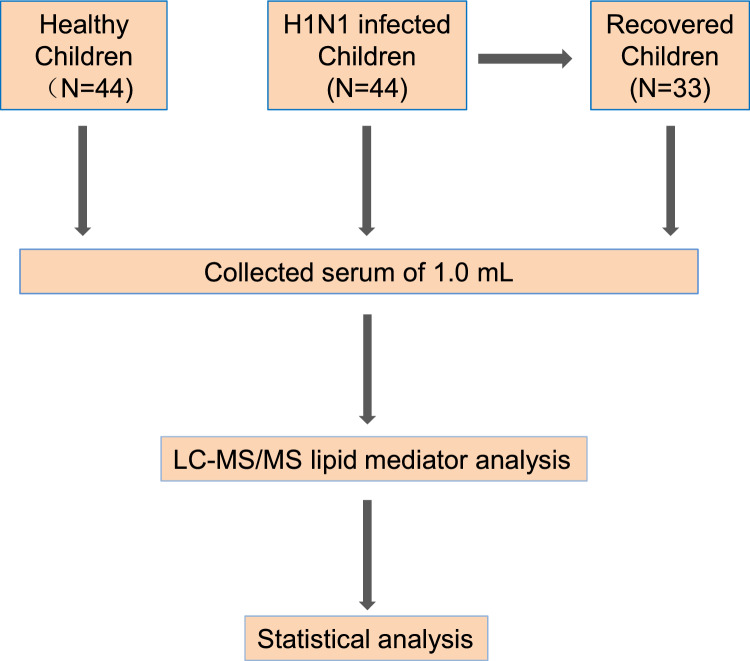
Study design. Flow chart showing the steps of this study.

We collected blood samples from 88 children, including 44 H1N1 children and 44 healthy age- and sex-matched children, from Shaoxing Shangyu Maternal and Child Health Care Hospital (Fig. 1). All H1N1 influenza cases were confirmed using real-time reverse transcription polymerase chain reaction (RT‒PCR), and diagnosed as mild symptoms based on the Diagnosis and Treatment Protocol for H1N1 influenza virus of the National Health Commission of China^16^. Among the H1N1 children, 36 returned for physical examination after recovery, and blood samples were also collected. All the H1N1 children were tested for other viruses, including coronavirus, adenovirus, influenza B, and respiratory syncytial virus. Only the children tested positive for H1N1 influenza virus and not other viruses were included in this study. The H1N1 children showed mild symptoms, such as fever (body temperature ≥ 37.2 °C) and cough, but no severe symptoms. The healthy children who did not experience any cold or flu symptoms, such as fever and cough, after 1 week were chosen for physical examination (Table 1). Blood tests revealed that white blood cell (WBC), lymphocyte (LYM), and platelet (PLT) counts were lower in H1N1 and recovered children than in healthy children, while neutrophil (NEUT) counts were greater in the H1N1 group and decreased in the recovered group. The serum samples were analyzed using a targeted LC‒MS/MS acquisition strategy and each lipid mediator was quantified based on its calibration curve generated by the synthesized standard. To monitor the data acquisition process, we interspersed quality control (QC) samples during mass spectrometry data acquisition, which contained a small portion of the serum samples from all samples in this study.

**Table 1:** Patient demographics and hematological and biochemical variables.

Patient characteristics	Healthy children (n = 44)	H1N1 children (n = 44)	Recovered children (n = 33)	ANOVA test		Bonferroni correction		
				F value	*P* value	Healthy versus H1N1	Healthy versus recovered	H1N1 versus recovered
Age (years)	3.86 (2.62)	4.57 (2.19)	4.73 (2.05)	1.5939	2.07E−01	ns	ns	ns
Gender								
Male	22 (50%)	22 (50%)	1 9 (58%)					
Female	22 (50%)	22 (50%)	14 (42%)					
WBC, 10^9^/L	7.77 (2.00)	5.90 (2.28)	5.20 (2.41)	14.2529	2.89E−06	***	****	ns
LYM, 10^9^/L	4.21 (1.58)	1.41 (0.92)	2.99 (1.37)	50.3941	1.64E−16	****	**	****
NEUT, 10^9^/L	2.94 (0.92)	3.90 (2.21)	1.71 (1.34)	17.1883	2.85E−07	*	***	****
HGB, g/L	125.11 (8.28)	122.68 (7.56)	120.00 (7.45)	3.9959	2.10E−02	ns	*	ns
PLT, 10^9^/L	310.09 (48.10)	202.23 (61.52)	244.38 (113.98)	22.8042	4.34E−09	****	*	ns
Fever	0 (0%)	44 (100%)	0 (0%)					
Cough	0 (0%)	44 (100%)	3 (9.1%)					

One-way ANOVA was applied to the healthy, H1N1 and recovered children. If differences among three groups were found, the Bonferroni correction was used for multiple comparisons, where *P value < 0.05, **P value < 0.01, and ***P value < 0.001. Continuous variables are given as the mean (SD), and categorical variables are given as the number of patients and percentage, n (%).

*WBC* white blood cell, *LYM* lymphocyte, *NEUT* neutrophil, *HGB* hemoglobin, *PLT* platelet.

### Differences of LM profiles between healthy controls and H1N1 children

**Figure 2:**
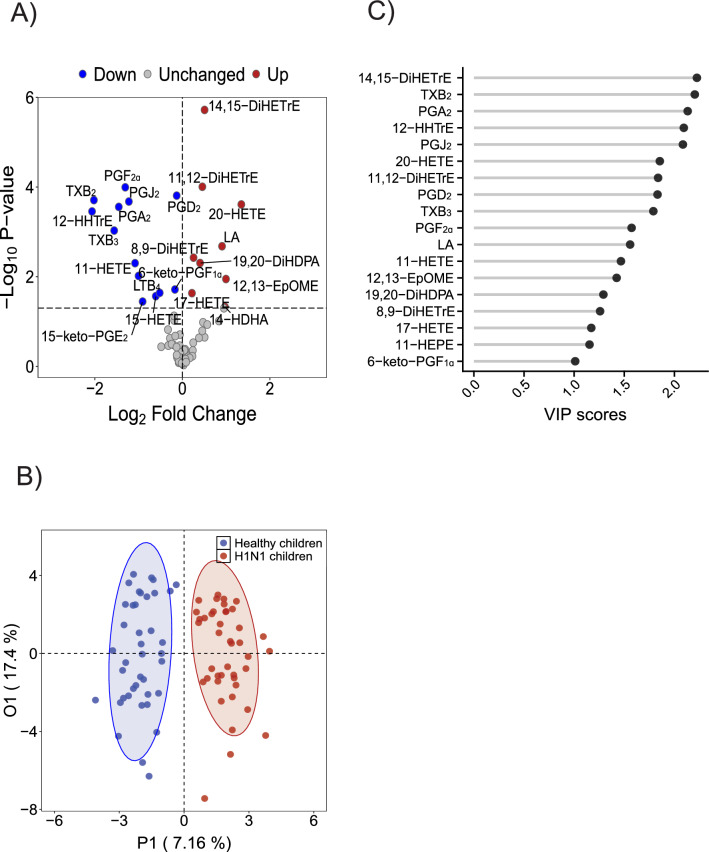
Serum LM remodeling after infection with H1N1 influenza virus in children. (**A**) Volcano plot of lipid mediators with the fold change (H1N1 children vs. healthy children) to the *P* value (Student’s t- test); (**B**) OPLS-DA score plots showing a separation between H1N1 children and healthy controls; (**C**) VIP scores of the LMs. A VIP greater than 1 highlights the most relevant indicators for identifying the two groups.

We identified 64 LMs in serum across all samples and determined the concentrations of LMs derived from various precursors, including n-6 PUFAs, n-6 linoleic acid (LA) and AA, as well as n-3 PUFAs, EPA and DHA. The QC samples showed good correlation and reproducibility in a pairwise comparison matrix (Suppl. Fig. 1). The relative standard deviation (RSD, %) of the peak areas of internal standards across all the QC samples (Suppl. Table 1) showed that the RSD was less than 15% for each internal standard, indicating that repeatability during LC‒MS/MS was acceptable. Our platform covers a broad range of oxidized fatty acid metabolites from diverse precursors and measures LMs at picomolar concentrations. We found that 22 lipid species were significantly changed in the blood of H1N1 children (*P* < 0.05) (Suppl. Table 2), compared with healthy controls. Eicosanoids and octadecanoids (derived from LA) were the major LMs altered after H1N1 influenza virus infection (Fig. 2A). In addition, using orthogonal partial least squares-discriminant analysis (OPLS-DA), we showed that H1N1 children and healthy controls were well separated based on serum LMs (Fig. 2B). The variable importance in projection (VIP) scores revealed 18 LMs that most strongly contributed to the observed separation (Fig. 2C). Notably, most LMs identified by VIP were identical to those identified by univariate analysis, as shown in Fig. 2A.

### The levels of serum LMs were restored after children recovered from H1N1 influenza

**Figure 3:**
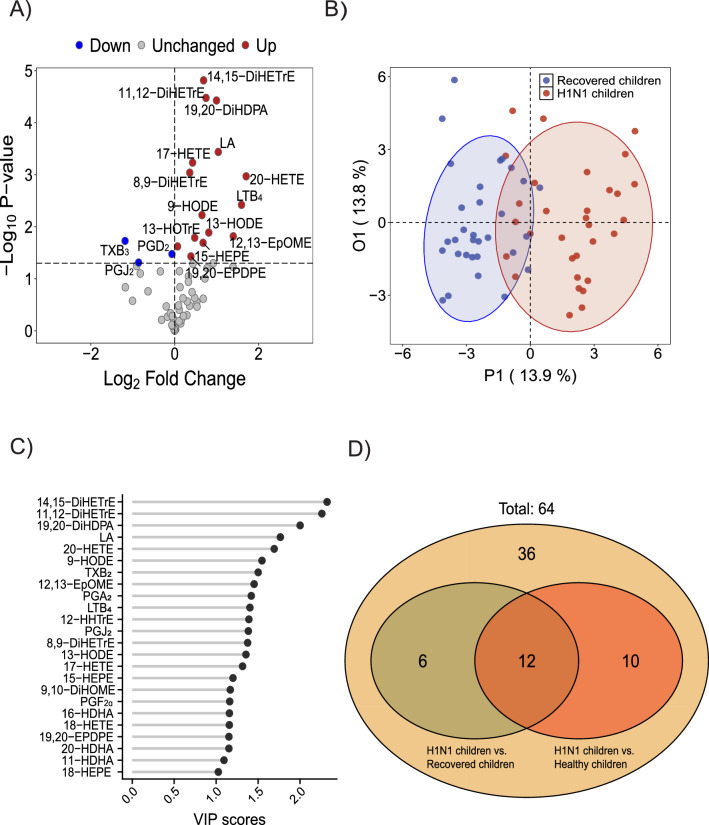
Serum LM alteration from H1N1 influenza to recovery in children. (**A**) Volcano plot of LMs, with the fold changes (H1N1 children versus recovered children) to the *P* value (paired Student’s t- test); (**B**) OPLS-DA score plots showing a separation between H1N1 and recovered children; (**C**) VIP scores of the LMs. A VIP greater than 1 highlights the most relevant indicators for identifying the H1N1 and recovered groups; (**D**) Venn diagram of significantly different LMs between the H1N1 and the healthy groups and between the H1N1 and recovered groups.

Furthermore, we used a targeted LC‒MS/MS method to measure serum LM levels after the children recovered from H1N1 influenza virus infection (Fig. 3A and Suppl. Table 3). OPLS-DA (Fig. 3B) revealed a partial separation between the recovered and H1N1 groups; the VIP scores (Fig. 3C) indicated that most of LMs whose levels were found to strongly contribute to the separation in OPLS-DA also showed statistically significant differences in their levels in paired Student’s t tests. To determine which LMs were restored to normal, we mapped the LM profiles of children across the healthy, H1N1 and recovery groups and revealed that the levels of 12 common LM species significantly differed between the healthy and H1N1 groups and between the H1N1 and recovered groups. (Fig. 3D).

**Figure 4:**
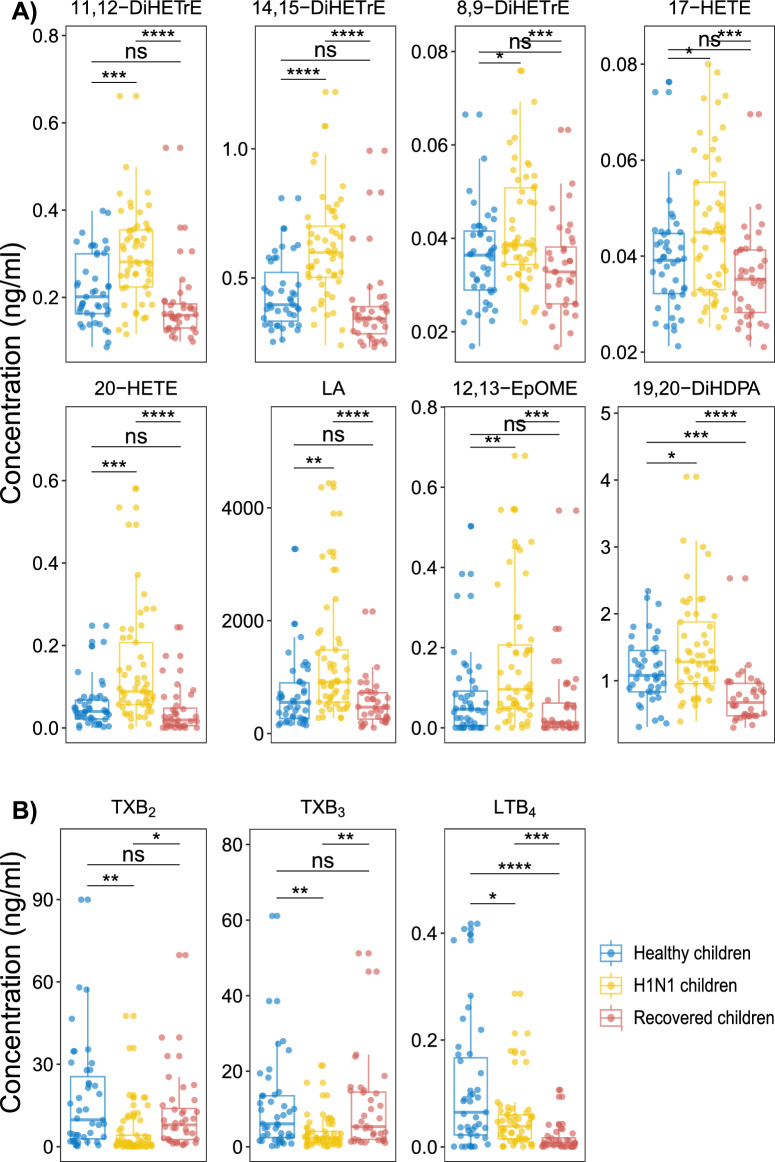
Temporal changes in serum LMs from healthy status to H1N1 influenza and then from H1N1 influenza to recovery in children. One-way ANOVA was applied to the healthy, H1N1, and recovered children. If a difference among three groups was found, the Bonferroni correction was used for multiple comparisons, where *P value < 0.05, **P value < 0.01, and ***P value < 0.001. Blue dot, healthy children; yellow dot, H1N1 children; red dot, recovered children.

**Figure 5:**
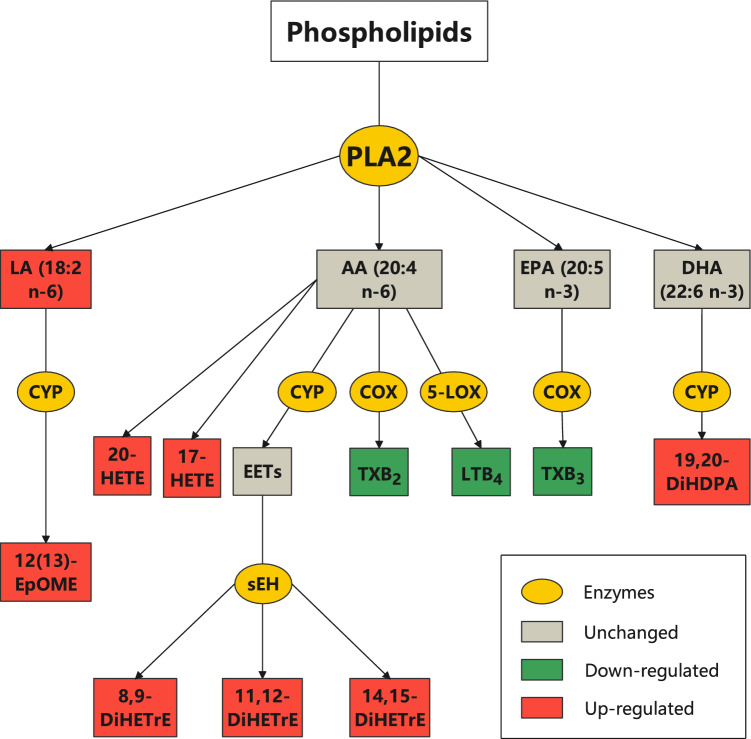
A LM metabolic pathway scheme highlighting the alterations in LA-, AA-, and EPA-derived LMs in H1N1 children compared with healthy and recovered children. *PLA2* phospholipase A2, *CYP* cytochrome P450, *COX* cyclooxygenase, *5-LOX* 5-lipoxygenase, *sEH* soluble epoxide hydrolase.

To further analyze the dynamic changes in relevant LM species in healthy, H1N1 and recovered individuals, we applied partial least squares-discriminant analysis (PLS-DA) to healthy, H1N1, and recovered children (Suppl. Fig. 2). The three groups partially overlapped and the VIP scores showed the relevant LMs for separating the three groups. Furthermore, one-way ANOVA was applied to the healthy, H1N1, and recovered children. We found that 25 LM species showed significant differences among these three groups (Suppl. Table 4), among which 11 LM species were partially or completely restored to normal after recovery, except for leukotriene B4 (LTB_4_) (Fig. 4). For these 11 LMs species, cytochrome P450 (CYP) enzyme-derived 11, 12-dihydroxyeicosatrienoic acids (11,12-DiHETrEs), 14,15-DiHETrE, 8,9-DiHETrE, 17-hydroxyeicosatetraenoic acid (17-HETE), and 20-HETE from AA, 12(13)- epoxyoctadecamonoenoic acid (12,(13)EpOME), 9,(10)-EpOME from LA, and LA, were increased in H1N1 children compared with the healthy and recovered groups, whereas cyclooxygenase (COX)-derived thromboxane B2 (TxB_2_) from AA and TxB_3_ from EPA were decreased in H1N1 children and then returned to normal after recovery. Interestingly, the level of the 5-LOX derived metabolite LTB_4_ from AA remained low in the recovered group. An LMs metabolic pathway scheme (Fig. 5) highlighted differences in LM biosynthetic pathways in H1N1 children compared with the healthy and recovered groups. This analysis demonstrated that most LMs, which were altered during H1N1 influenza, contained upregulated CYP-derived products from AA and LA and downregulated products from COX enzymes. These results suggest that these enzymes exhibit a unique pattern in the activity during H1N1 influenza virus infection in children.

### Correlations between hematological variables and LM levels

Before the correlation analysis was performed, the normality of each LM species was examined using the Shapiro–Wilk normality test. We found that all the LM levels were not normally distributed; therefore, Spearman correlation was applied to examine the relationships between hematological variables and LM levels. The LMs that correlated significantly with hematological variables are listed in Suppl. Table 5, with correlation coefficients (r) and *P* values < 0.05. The range of the r values of the LMs ranges from − 0.413 to 0.487, indicating weak to moderate associations between hematological variables and LM concentrations. Among the 11 LM species identified during the progress from health to H1N1 influenza and back to recovery, NEUT count was positively correlated with LTB_4_ (r = 0.412), 19, 20-DiHDPA (r = 0.388), 14,15-DiHETrE (r = 0.386), 20-HETE (r = 0.369), 11,12-DiHETrE (r = 0.351), 12, 13-EpOME (r = 0.265), LA (r = 0.219), and 17-HETE (r = 0.203) but negatively correlated withTxB_3_ (r = − 0.239) and TxB_2_ (r = -0.265); PLT count was positively correlated with TxB_2_ (r = 0.303), TxB_3_ (r = 0271) and LTB_4_ (r = 0.244) but negatively correlated with 20-HETE (r = − 0.181), 14,15-DiHETErE (r = − 0.182), and 12,13-EpOME (r = -0.219); LYM count was positively correlated with TxB_2_ (r = 0.487), and TxB_3_ (r = 0.361) but negatively correlated with 19,20-DiHDPA (r = − 0.191), 8,9-DiHETrE (r = − 0.228), 17-HETE (r = − 0.216), 20-HETE (r = − 0.385), 12,13-EpOME (r = − 0.278), 11,12-DiHETrE (r = − 0.360), LA (r = − 0.331), and 14,15-DiHETrE (r = − 0.413); WBC count was positively correlated with LTB_4_ (r = 0.395) and 19.20-DiHDPA (r = 0.220); Hemoglobin (HGB) was positively correlated with 12,13-EpOME (r = 0.201) and 11,12-DiHETrE (r = 0.182).

## Discussion

In this study, we applied a targeted LC‒MS/MS platform to systematically investigate the profile of serum LMs in H1N1 children. We demonstrated that eicosanoids from AA and octadecanoids from LA were dysregulated in H1N1 children. Furthermore, the level of 11 LM species were fully or partially restored to healthy levels after recovery. Taken together, these findings suggest that disruptions in the biosynthesis of LMs, especially eicosanoids, occur during H1N1 influenza virus infection and clearance in children.

Bioactive LMs play a crucial role in the induction and resolution of inflammation during influenza virus infection^5,6,12^. Controlled immune responses to infection and injury involve complex molecular networks and often opposing actions^12^. Irun et al.^17^ revealed that the levels of serum LMs, such as prostaglandin E_2_ (PGE_2_), and resolving E1, 14-hydroxy docosahexaenoic acid (14-HDHA), were increased in the patients with severe COVID-19 caused by SARS-CoV-2. Palmas et al.^14^ reported that the levels of lipoxygenase pathway-related mediators, such as LTB_4_ and RvD1, were lower in critically ill COVID-19 patients than in those with severe disease. In mouse model infected with the influenza virus A/Puerto Rico/8/34 (PR8/H1N1), PGs in bronchoalveolar lavage increased in the early inflammation phase and decreased thereafter; H1N1 virus-infected mice also exhibited decreased levels of LOX-derived metabolites and increased levels of CYP pathway-related metabolites^12^. Our study showed that the levels of the CYP pathway-derived products DiHETrEs, 17-HETE, 20-HETE, 12,13-EpOME, and 19,20-DiHDPA increased in H1N1 children with mild symptoms and decreased to basal levels after recovery. These results are consistent with a previous study in which CYP pathway-derived metabolites were increased in patients with severe H1N1 influenza-induced pneumonia compared with healthy adult individuals^15^. Moreover, the levels of COX pathway products TxB_2_ and TxB_3_ were reduced in H1N1 children but returned to normal levels after recovery. The exception is LTB_4_, which is biosynthesized by leukocytes during inflammation^18^; LTB_4_ level remained low in H1N1 influenza and further decrease even after recovery. Our results suggested that during H1N1 influenza, serum LMs, especially eicosanoids, are tightly regulated to counterbalance the severity of inflammation and maintain the physiological functions.

TxB_2_ and TxB_3_ are thromboxanes that are mainly biosynthesized from AA and EPA, respectively, with catalysis by thromboxane synthase in platelets. TxA_2_ and TxA_3_ are active products of platelets and are quickly hydrolyzed to the inactive forms TxB_2_ and TxB_3_ nonenzymatically^19–21^. Local TxA_2_ and TxA_3_ levels are critical for the regulation of systemic blood pressure and thrombogenesis^22^. The levels of TxA_2_ and TxA_3_ are positively correlated with the PLT count. The lower PLT count in H1N1 children is considered to be associated with the reduction in thromboxane levels. After the children recovered from H1N1 influenza virus infection, the levels of these bioactive compounds were restored, as the PLT count was restored to the normal level.

LTB_4_, which activates leukocytes and prolongs their survival, is the most important leukotriene in acute inflammatory responses. Additionally, LTB_4_ is a powerful chemoattractant for neutrophils and macrophages and stimulates leukocyte adhesion to the vascular endothelium by upregulating integrin expression^23,24^. LTB_4_ biosynthesis is initiated by the 5-LOX-mediated conversion of arachidonic acid to leukotriene A4 (LTA_4_), after which LTA_4_ is subsequently converted to LTB_4_. The enzyme LTA_4_ hydrolase converts LTA_4_ to LTB_4_ mainly in white blood cells, including neutrophils, macrophages, and monocytes^25^. Our study indicated that LTB_4_ was positively correlated with the NEUT, PLT and WBC counts. The reduction in LTB_4_ in H1N1 children compared to healthy controls might be linked to the total reduction in the WBC count, although the neutrophil count was greater. A further reduction in the WBC count might lead to a decreased LTB_4_ level even after recovery from H1N1 influenza.

Inflammation is an essential host defense mechanism that is necessary for protection against pathogens, microorganisms and viruses. However, excessive and nonresolving inflammatory responses are damaging to the host^6,10^. H1N1 influenza virus infection triggers an excessive inflammatory response in hosts and, if untreated, can lead to influenza-induced pneumonia or other complications, including sepsis syndrome and respiratory distress syndrome^26^. Patients that overlapped between H1N1 and recovered children might have different dynamic process for inflammation to resolution^27,28^, leading to some recovered children still unresolved. During the process of inflammation, LMs from PUFAs, including AA, LA, EPA, and DHA, are synthesized and play a crucial role in controlling inflammation and combating infectious organisms. In the early stage of inflammation, LTs and PGs are important for promoting the immune response following tissue injury^7,8^. During the resolution of inflammation, SPMs with anti-inflammatory and proresolving effects are produced and have potent immunoregulatory effects^12^. In influenza virus infection, a DHA-derived resolvin, 17-HDHA, upregulates the production of neutralizing antibodies to the virus^13^, whereas PD family members inhibit influenza virus replication via RNA export machinery^11^. Notably, during H1N1 influenza, the levels of vicinal diols, such as 11,12-DiHETrE, 14,15-DiHETrE, 8,9-DiHETrE from AA, and other metabolites produced via the CYP and soluble epoxide hydrolase (sEH) pathways, increase in children with disease progression of H1N1 influenza. Epoxyeicosatrienoic acids (EETs), which are CYP intermediate products, are anti-inflammatory, analgesic, antifibrotic, and antihypertensive agents that act in both autocrine and paracrine manners^29^. The end metabolites of these compounds, vicinal diols, might be used as potential biomarkers for inflammatory and related diseases.

In conclusion, our study revealed different serum LM profiles in children with H1N1 influenza virus infection associated with inflammatory responses. We identified a number of LM species that were altered by H1N1 influenza and restored after spontaneous recovery in children. These findings shed light on the pathogenesis of H1N1 influenza in children, and provided foundations for potential therapeutic tools to treat this disease.

## Methods

### Study subjects

Forty-four H1N1 children and forty-four age- and sex- matched healthy control children aged 1–10 years were included in this study from Shaoxing Shangyu Maternal and Child Health Care Hospital. H1N1 influenza virus was confirmed using direct immunofluorescence assays according to the Diagnosis and Treatment Protocol for H1N1 influenza virus of the National Health Commission of China^16,30^. Oropharynx swabs were collected, and the swabs were tested for various viruses, including adenovirus (ADV), influenza A (FLUA), influenza B (FLUB) and respiratory syncytial virus (RSV), using colloidal gold methods. For influenza A-positive samples, H1N1 was further confirmed via real-time RT‒PCR^31,32^. Briefly, total viral RNA from throat swab samples was extracted using the QIAamp Viral RNA Mini Kit (Qiagen, Valencia, CA) according to the manufacturer’s protocol and stored at − 80 °C, RT‒PCR was performed with a one-step PrimeScript^®^ RT‒PCR reagent (Takara, Dalian, China). The final optimized 50 μl reaction mixture consisted of 5 μl of RNA, 25 μl of 2 × One Step RT‒PCR Buffer, 1 μl of TaKaRa EX Taq HS (5 U/μl), 1 μl of Prime-Script RT Enzyme Mix, 1 μl of forward primer (40 μM), 1 μl of reverse primer (40 μM), and 0.5 μl of probe (20 μM) for H1N1 influenza virus (Suppl. Table 6). The thermal cycling parameters were as follows: cDNA synthesis at 42 °C for 30 min; denaturation at 95 °C for 5 min; and 40 cycles at 95 °C for 10 s and 60 °C for 45 s. The fluorescence was recorded at 60 °C on an ABI 7500 real-time PCR system (Applied Biosystems, Foster City, CA). Only those with H1N1 influenza virus infections and no coinfections were included in this study. The healthy children were chosen based on the absence of cold or flu symptoms, such as fever (body temperature ≥ 37.2 °C) or cough, within 1 week. Human serum samples were collected after an overnight fast. Among the H1N1 children, thirty-six returned to the hospital for physical examination after recovery for approximately 5–7 days and mostly showed no symptoms of fever, excluding three patients who still had a cough. The serum samples for the recovery group were collected and used for analysis. The study conformed to the Declaration of Helsinki and was approved by the Medical Ethics Committee of Shaoxing Shangyu Maternal and Child Health Care Hospital (LL-2023-07-05). Written informed consent was obtained from a parent and/or legal guardian for study participation.

### LM extraction

All LM standards were purchased from the Cayman Chemical Company (Ann Arbor, MI, USA). HLB SPE cartridges were purchased from Anbocis Technology (Beijing, China). All HPLC-grade solvents were acquired from Sigma–Aldrich (St. Louis, MO, USA) or Merck & Co., Inc. (Kenilworth, NJ, USA). Before lipid extraction, human serum samples were thawed on ice. Small aliquots (20 μl) of each human serum sample were pooled as QC samples and thoroughly mixed for 3 min. Aliquots of 100 μl of thawed human serum and QC samples were used for sample preparation. A mixture of deuterium-labeled internal standards was added to each sample, followed by the addition of 1.5 mL of cold methanol (MeOH). The samples were vortexed for 5 min and stored at − 20 °C overnight. The cold samples were centrifuged at 14,000 rpm for 5 min, and the supernatant was then transferred to a new tube. The samples were evaporated under nitrogen to volume of 50 μl, after which 1 mL of acidified H_2_O (pH 3.5) was added to each sample before HLB solid phase extraction (SPE) as previously described^33^. In brief, the acidified samples were loaded onto an HLB SPE column, washed with water and hexane, and eluted with methyl formate. The methyl formate fractions were collected, dried under nitrogen, and reconstituted in 100 μL of MeOH:H_2_O (2:3, v/v). The samples were centrifuged at 20,000 rpm, and 80 μl of each supernatant was subjected to LC–MS/MS analysis.

### LC–MS/MS for LMs

LC–MS/MS for LMs has been reported previously^33^. Briefly, LMs were analyzed by LC–MS/MS using a Shimadzu LC-20ACXR (Shimadzu, Kyoto, Japan) and Sciex 5500 triple quadruple mass spectrometer (Sciex, MA, USA) with heated electrospray ionization (ESI). A Phenomenex Kinetex C18 column (100 × 2.1 mm, 2.6 μm) was maintained at 40 °C. Solvent A was composed of 100% H_2_O with 0.1% formic acid, and solvent B was composed of 100% acetonitrile (ACN) with 0.1% formic acid. A gradient was applied with 35% B at 0 min, increased to 50% B until 4.0 min, increased to 95% B at 9.0 min, maintained at 95% B until 11.5 min, returned to 35% B at 12.0 min, and maintained at 35% B until 14.0 min, with a flow rate of 0.3 mL/min. The ESI–MS instrument was operated in negative mode with a voltage of − 4500 V, a temperature of 500 °C, a curtain gas at 30 psi, GS1 at 40 psi, and GS2 at 40 psi. The mass spectrometry parameters for each lipid were optimized, and a scheduled multiple reaction monitoring (MRM) strategy was applied. The retention time, specific MRM transitions, and corresponding internal standards for each lipid mediator are listed in Suppl. Table 7. We used different deuterated standards to normalize individual lipid mediators according to their structural similarities and chromatographic behaviors, i.e., retention times. Calibration curves for the LMs were generated using synthetic LM mixtures, including appropriate internal standard mixtures (d4-LTB_4_, d5-LXA_4_, d4-PGE_2_, d5-RvD_2_, and d8-5-HETE), with a linear regression coefficient of determination (R^2^) ≥ 0.99. Quantitation was performed using MutliQuant software (Sciex, MA, USA). During LC–MS/MS, QC samples were interspersed after 20 injections, with 6 QC samples positioned at the beginning and end of the analysis.

### Statistical analysis

The *P* values were calculated by Student’s t test or paired Student’s t test, and values greater than 0.05 indicated statistically significant differences. One-way ANOVA was applied to three groups. If a significant difference among three groups was found, the Bonferroni correction was used for multiple comparisons. OPLS-DA and PLS-DA were performed to identify LMs that discriminate between different groups. VIP scores allowed the classification of variables according to their explanatory power. The predictors with large VIPs were the most relevant. The normality of hematological variables and the concentration of each LM were examined by the Shapiro–Wilk normality test. Pearson correlation was used if the data followed a normal distribution, or Spearman correlation was applied for the correlation analysis, with *P* < 0.05 considered to indicate statistical significance. The data were analyzed and plots were generated using R version 4.2.2.

### Supplementary Information

Supplementary Information.

## Data Availability

The datasets generated during the current study are available from the corresponding authors upon reasonable request.
