# Supplementary material for: Profiling lipid mediators in serum from children with H1N1 influenza
Source: Sci Rep. 2024 Jul 2;14:15186. doi: 10.1038/s41598-024-66190-y (PMC11219859; doi:10.1038/s41598-024-66190-y)
Supplement: Supplementary file 1 — Supplementary Information. [file 41598_2024_66190_MOESM1_ESM.docx]

**Profiling lipid mediators in serum from children with H1N1 influenza**

Weijun Chen^1^, Yitao Gu^2^, Yongjun Ma^2^, Lele Dong^3^，Liangxuan Pan^3^, Chai Ji^1^, Lanlan Guo^2^, Lianxin Qi^4^, Yuanyuan Zhang^5*^, Fei Gao^3*^

1. Department of Child Health Care, Children's Hospital, Zhejiang University School of Medicine, National Clinical Research Center for Child Health, Hangzhou, China

2. Department of Pediatrics，Shaoxing Shangyu Maternal and Child Health Care Hospital, Shangyu District, Shaoxing, China,

3. Durbrain Medical Laboratory, Hangzhou, China.

4. Department of Clinical Laboratory，Shaoxing Shangyu Maternal and Child Health Care Hospital, Shangyu District, Shaoxing, China,

5. Department of Pulmonology, Children's Hospital, Zhejiang University School of Medicine, National Clinical Research Center for Child Health, Hangzhou, China

* Correspondence:

Yuanyuan Zhang, E-mail: [chzyy@zju.edu.cn](mailto:chzyy@zju.edu.cn)

Fei Gao, E-mail: [fei.gao@durbrain.com](mailto:fei.gao@durbrain.com)

**Supplementary Figure Legends**

**Supplementary Figure 1**. Correlation analysis of the quality control (QC) samples.


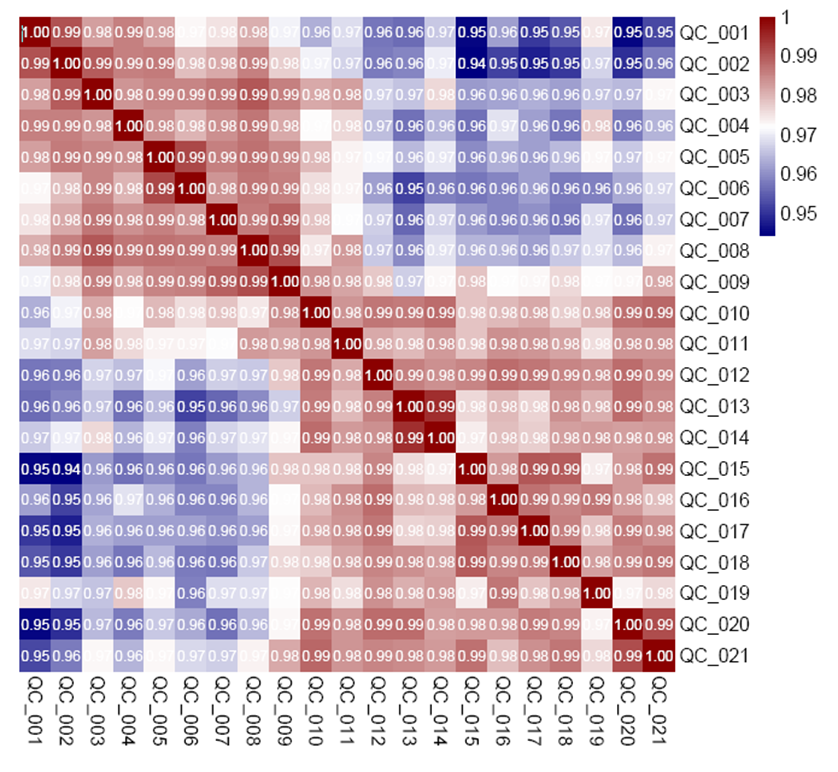


**Supplementary Figure 2**. A) A PLS-DA score plot showing a separation across healthy, H1N1 and recovered children; B). VIP scores of the LMs. VIP larger than 1 highlights the most relevant indicators for identifying the different groups.


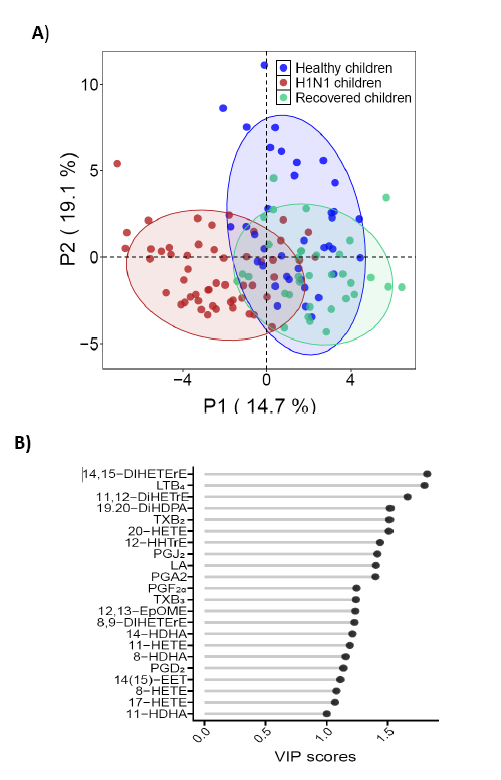


**Supplementary Tables**

**Supplementary Table1**. Relative standard deviations (RSD, %) of deuterium labeled internal standards in quality control (QC) samples across the LC–MS/MS analysis (n=21)

| **Internal**  **standards** | **d4-PGE_2_** | **d8-5s-HETE** | **d4-LTB_4_** | **d4-9-HODE** | **d4-9,10-diHOME** |
| --- | --- | --- | --- | --- | --- |
| RSD (%) | 10.3% | 14.0% | 4.9% | 7.2% | 5.1% |

**Supplementary Table 2.** LM profiles in serum from H1N1 and healthy children by LC–MS/MS.

| **Lipid mediator (ng/mL)** | **H1N1** | **Healthy** | **P value*** |
| --- | --- | --- | --- |
| 14,15-DiHETrE | 0.62 ± 0.2 | 0.44 ± 0.13 | 1.91E-06 |
| 11,12-DiHETrE | 300.88 ± 104.07 | 219.16 ± 80.77 | 9.88E-05 |
| PGF_2α_ | 42.68 ± 44.57 | 105.43 ± 88.67 | 1.02E-04 |
| PGD_2_ | 27.6 ± 1.3 | 30.18 ± 3.95 | 1.56E-04 |
| TXB_2_ | 4.19 ± 9.15 | 17 ± 19.14 | 1.96E-04 |
| PGJ_2_ | 14.06 ± 11.48 | 32.79 ± 28.78 | 2.11E-04 |
| 20-HETE | 137.41 ± 130.2 | 53.99 ± 54.98 | 2.45E-04 |
| PGA_2_ | 11.75 ± 11.9 | 32.17 ± 32.29 | 2.78E-04 |
| 12-HHTrE | 2.94 ± 6.21 | 12.28 ± 14.87 | 3.51E-04 |
| TXB_3_ | 3.47 ± 4.38 | 10.2 ± 11.84 | 9.37E-04 |
| LA | 1283.43 ± 1084.12 | 684.49 ± 597.86 | 2.10E-03 |
| 8,9-DiHETrE | 42.59 ± 11.78 | 35.67 ± 9.82 | 3.77E-03 |
| 19.20-DiHDPA | 1.53 ± 0.7 | 1.16 ± 0.49 | 4.96E-03 |
| 11-HETE | 0.79 ± 1.07 | 1.66 ± 1.68 | 5.00E-03 |
| LTB_4_ | 55.04 ± 60.69 | 110.13 ± 121.14 | 9.62E-03 |
| 12,13-EpOME | 153.64 ± 163.3 | 77 ± 106.52 | 1.13E-02 |
| 6-keto-PGF_1α_ | 15.6 ± 2.6 | 17.55 ± 4.66 | 1.93E-02 |
| 11-HEPE | 50.93 ± 32.23 | 72.88 ± 53.06 | 2.30E-02 |
| 17-HETE | 45.82 ± 14.51 | 39.36 ± 11.38 | 2.33E-02 |
| 15-HETE | 107.01 ± 52.66 | 162.97 ± 152.85 | 2.72E-02 |
| 15-keto-PGE_2_ | 30.7 ± 19.52 | 57.6 ± 79.34 | 3.59E-02 |
| 14-HDHA | 6.99 ± 10.22 | 3.56 ± 4.61 | 4.73E-02 |
| 8-HDHA | 3.75 ± 5.46 | 1.94 ± 2.51 | 5.06E-02 |
| 13-HDHA | 22.11 ± 3.23 | 25.21 ± 10.71 | 7.48E-02 |
| 5,6-DiHETrE | 97.35 ± 32.24 | 110.68 ± 40.65 | 9.47E-02 |
| 13,14-dihydro-15-keto PGF_2α_ | 38.3 ± 64.71 | 21.46 ± 13.15 | 9.75E-02 |
| 2,3-dinor-11β-PGF_2α_ | 36.39 ± 37.11 | 26.22 ± 16.95 | 1.04E-01 |
| 11-HDHA | 0.92 ± 1.1 | 0.62 ± 0.61 | 1.17E-01 |
| RvD1 | 10.54 ± 3.87 | 13.24 ± 11.53 | 1.52E-01 |
| 14(15)-EET | 185.06 ± 161.99 | 135.02 ± 168.68 | 1.62E-01 |
| 18-HEPE | 37.28 ± 3.25 | 38.78 ± 6.83 | 1.96E-01 |
| PGB_2_ | 33.03 ± 11.44 | 36.95 ± 16.95 | 2.11E-01 |
| 1-Mar | 17.17 ± 24.39 | 12.07 ± 12.69 | 2.25E-01 |
| 20-HDHA | 1.27 ± 1.47 | 0.97 ± 0.66 | 2.32E-01 |
| 16-HETE | 1.19 ± 0.74 | 1.45 ± 1.21 | 2.36E-01 |
| 18-HETE | 0.64 ± 0.46 | 0.54 ± 0.44 | 2.97E-01 |
| 16-HDHA | 0.32 ± 0.22 | 0.38 ± 0.32 | 3.19E-01 |
| 5-HEPE | 224.05 ± 127.9 | 258.65 ± 194.36 | 3.31E-01 |
| 9,10-DiHOME | 5.13 ± 2.99 | 6.4 ± 8.19 | 3.43E-01 |
| 9-HETE | 76.52 ± 46.78 | 95.59 ± 122.72 | 3.45E-01 |
| 5,15-DiHETE | 37.88 ± 8.79 | 39.51 ± 7.21 | 3.46E-01 |
| 12-HETE | 1.16 ± 1.22 | 1.62 ± 3.09 | 3.65E-01 |
| 19,20-EPDEP-2 | 0.62 ± 0.25 | 0.68 ± 0.36 | 3.73E-01 |
| 5,6-DiHETE | 69.76 ± 20.14 | 66.77 ± 13.29 | 4.15E-01 |
| 8(9)-EET | 32.88 ± 5.55 | 31.85 ± 6.59 | 4.35E-01 |
| 5(6)-EET | 223.2 ± 220.61 | 189.22 ± 188.21 | 4.41E-01 |
| 7-HDHA | 18.52 ± 2.74 | 19.16 ± 5.37 | 4.91E-01 |
| 19,20-EPDPE | 0.48 ± 0.3 | 0.53 ± 0.41 | 5.59E-01 |
| 9-HODE | 2.9 ± 1.42 | 2.68 ± 2.1 | 5.59E-01 |
| 8-HETE | 8.43 ± 12.89 | 7.19 ± 8.44 | 5.96E-01 |
| 13-HODE | 1.91 ± 1.45 | 1.74 ± 1.69 | 6.13E-01 |
| 12,13-DiHOME | 5.45 ± 3.57 | 5.93 ± 5.32 | 6.26E-01 |
| 4-HDHA | 31.14 ± 7.51 | 32.03 ± 10.19 | 6.44E-01 |
| 12-HEPE | 99.18 ± 105.28 | 88.4 ± 115.57 | 6.51E-01 |
| 17-HDHA | 75.57 ± 49.65 | 81.64 ± 76.35 | 6.62E-01 |
| 13-HOTrE | 144.16 ± 73.08 | 137.02 ± 93.97 | 6.94E-01 |
| 15-HEPE | 135.22 ± 100.16 | 142.8 ± 145.13 | 7.78E-01 |
| 15-Oxo ETE | 102.9 ± 109.8 | 96.99 ± 112.19 | 8.04E-01 |
| 5-HETE | 56.07 ± 24.91 | 57.4 ± 28.49 | 8.18E-01 |
| ARA | 1.41 ± 1.68 | 1.49 ± 1.85 | 8.30E-01 |
| 5-iPF_2α_-VI | 2.12 ± 0.6 | 2.15 ± 0.49 | 8.48E-01 |
| 16(17)-EpDPE | 20.71 ± 4.16 | 20.55 ± 4.21 | 8.60E-01 |
| Lipoxin A_4_ | 12.61 ± 4.16 | 12.51 ± 3.91 | 9.06E-01 |
| DHA | 4.97 ± 5.05 | 4.87 ± 5.45 | 9.33E-01 |

Data are means ± SD (N=44).

**Supplementary Table 3.** LM profiles of serum from H1N1 and recovered children by LC–MS/MS.

| **Lipid mediator (ng/mL)** | **H1N1** | **Recovery** | **P value** |
| --- | --- | --- | --- |
| 14,15-DiHETrE | 0.61 ± 0.2 | 0.38 ± 0.16 | 1.54E-05 |
| 11,12-DiHETrE | 295.59 ± 108.44 | 176.19 ± 85.32 | 3.35E-05 |
| 19.20-DiHDPA | 1.49 ± 0.82 | 0.75 ± 0.41 | 3.75E-05 |
| LA | 1051.93 ± 673.19 | 512.03 ± 398.47 | 3.65E-04 |
| 17-HETE | 48.06 ± 15.75 | 35.78 ± 9.79 | 5.85E-04 |
| 8,9-DiHETrE | 42.69 ± 11.77 | 33.3 ± 9.65 | 9.14E-04 |
| 20-HETE | 140.18 ± 137.27 | 43.05 ± 55.87 | 1.07E-03 |
| LTB_4_ | 50.66 ± 63.08 | 16.79 ± 24.87 | 3.79E-03 |
| 9-HODE | 2.98 ± 1.54 | 1.9 ± 1.48 | 5.97E-03 |
| 13-HODE | 2.11 ± 1.82 | 1.2 ± 0.97 | 1.29E-02 |
| 12,13-EpOME | 145.19 ± 167.81 | 55.14 ± 104.87 | 1.52E-02 |
| 13-HOTrE | 145.96 ± 98.06 | 104.92 ± 56.67 | 1.63E-02 |
| TXB_3_ | 4.47 ± 4.85 | 10.15 ± 12.04 | 1.87E-02 |
| 15-HEPE | 129 ± 107.97 | 80.75 ± 55.6 | 2.03E-02 |
| 18-HEPE | 37.19 ± 3.36 | 35.49 ± 2.97 | 2.39E-02 |
| PGD_2_ | 27.71 ± 1.31 | 29.02 ± 3.35 | 3.34E-02 |
| 19,20-EPDPE | 0.43 ± 0.22 | 0.33 ± 0.18 | 3.71E-02 |
| PGJ_2_ | 15.94 ± 12.56 | 28.97 ± 36.42 | 4.87E-02 |
| 11-HDHA | 0.88 ± 1.13 | 0.46 ± 0.4 | 5.02E-02 |
| 18-HETE | 0.67 ± 0.47 | 0.5 ± 0.31 | 5.15E-02 |
| TXB_2_ | 6.38 ± 10.79 | 11.94 ± 14.05 | 5.55E-02 |
| PGA_2_ | 14.05 ± 12.81 | 26.34 ± 35.18 | 5.68E-02 |
| 13,14-dihydro-15-keto PGF_2α_ | 40.82 ± 72.3 | 15.57 ± 14.53 | 5.90E-02 |
| 20-HDHA | 1.37 ± 1.64 | 0.8 ± 0.7 | 6.25E-02 |
| 7-HDHA | 18.54 ± 3.27 | 17.14 ± 2.39 | 7.14E-02 |
| 6-keto-PGF_1α_ | 15.77 ± 2.56 | 19.99 ± 13.37 | 7.21E-02 |
| 12-HHTrE | 4.23 ± 7.11 | 7.47 ± 8.73 | 7.91E-02 |
| 5-HETE | 59.63 ± 32.07 | 47.48 ± 21.15 | 9.00E-02 |
| 9,10-DiHOME | 5.78 ± 7.26 | 3.51 ± 2.93 | 9.85E-02 |
| 17-HDHA | 71.01 ± 45.27 | 56.63 ± 37.72 | 1.14E-01 |
| 5,6-DiHETE | 69.06 ± 19.17 | 63.29 ± 11.44 | 1.45E-01 |
| PGF_2α_ | 50.74 ± 46.81 | 114.81 ± 246.77 | 1.46E-01 |
| 11-HETE | 1.08 ± 1.33 | 1.68 ± 2.28 | 1.74E-01 |
| 12-HEPE | 89.13 ± 84.03 | 66.96 ± 48.81 | 1.80E-01 |
| Mar1 | 19.16 ± 28.87 | 13.8 ± 12.53 | 2.33E-01 |
| 19,20-EPDEP | 0.62 ± 0.28 | 0.54 ± 0.25 | 2.36E-01 |
| 14-HDHA | 6.38 ± 10.91 | 3.98 ± 4.52 | 2.41E-01 |
| 15-keto-PGE_2_ | 32.18 ± 21.6 | 64.66 ± 161.9 | 2.65E-01 |
| 16-HDHA | 0.31 ± 0.25 | 0.25 ± 0.16 | 2.72E-01 |
| 8-HETE | 9.85 ± 14.82 | 6.85 ± 8.6 | 2.76E-01 |
| 4-HDHA | 30.94 ± 7.93 | 29.05 ± 7.68 | 3.26E-01 |
| 12,13-DiHOME | 6.57 ± 7.92 | 5.13 ± 4.73 | 3.30E-01 |
| 15-HETE | 118.96 ± 60.67 | 136.85 ± 109.43 | 3.39E-01 |
| 8-HDHA | 3.34 ± 5.76 | 2.31 ± 2.74 | 3.43E-01 |
| RvD1 | 10.14 ± 3.81 | 9.42 ± 2.54 | 3.94E-01 |
| PGB_2_ | 33.46 ± 9.16 | 35.91 ± 18.65 | 4.60E-01 |
| DHA | 4.06 ± 4.61 | 3.32 ± 4.34 | 5.02E-01 |
| 14(15)-EET | 195.28 ± 156.74 | 169.03 ± 157.23 | 5.18E-01 |
| 5-iPF_2α_-VI | 2.04 ± 0.46 | 2.13 ± 0.51 | 5.21E-01 |
| 11-HEPE | 49.88 ± 30.38 | 56.3 ± 49.43 | 5.36E-01 |
| 13-HDHA | 21.6 ± 2.21 | 21.98 ± 3.68 | 5.98E-01 |
| 2,3-dinor-11β-PGF_2α_ | 34.91 ± 36.72 | 31.21 ± 18.34 | 6.09E-01 |
| ARA | 1.23 ± 1.75 | 1.53 ± 2.9 | 6.16E-01 |
| 9-HETE | 77.56 ± 48.37 | 72.12 ± 65.53 | 6.56E-01 |
| 12-HETE | 1.19 ± 1.28 | 1.08 ± 1.58 | 7.20E-01 |
| 15-Oxo ETE | 117.66 ± 111.07 | 123.24 ± 101.12 | 7.71E-01 |
| 16-HETE | 1.26 ± 0.79 | 1.31 ± 0.95 | 8.05E-01 |
| 5,6-DiHETrE | 108.75 ± 59.88 | 111.69 ± 48.66 | 8.29E-01 |
| 8(9)-EET | 32.22 ± 4.63 | 32.03 ± 7.26 | 8.92E-01 |
| 5,15-DiHETE | 38.8 ± 9.48 | 39.02 ± 6.08 | 9.15E-01 |
| 5-HEPE | 222.17 ± 112.88 | 225.01 ± 217.39 | 9.33E-01 |
| Lipoxin A_4_ | 13.25 ± 4.41 | 13.16 ± 4.11 | 9.42E-01 |
| 5(6)-EET | 190.15 ± 173.73 | 187.07 ± 175.04 | 9.45E-01 |
| 16(17)-EpDPE | 20.16 ± 2.36 | 20.16 ± 3.53 | 9.93E-01 |

Data are means ± SD (N=36).

**Supplementary Table 4**. F value and P value after One-way ANOVA analysis was applied to the healthy, H1N1 and recovered children.

| **Lipid mediator** | **F value** | **P value** |
| --- | --- | --- |
| Lipoxin A_4_ | 1.495 | 0.228007 |
| PGD_2_ | 8.115 | 0.000479 |
| PGF_2a_ | 3.357 | 0.037907 |
| TXB_2_ | 8.666 | 0.000294 |
| RvD1 | 3.277 | 0.040893 |
| LA | 11.465 | 2.61E-05 |
| 9,10-DiHOME | 1.545 | 0.217282 |
| 12,13-DiHOME | 0.069 | 0.933012 |
| 9-HODE | 2.282 | 0.106163 |
| 13-HODE | 2.028 | 0.135837 |
| ARA | 0.053 | 0.948517 |
| 5-HETE | 1.935 | 0.148543 |
| 12-HETE | 0.971 | 0.381531 |
| 15-HETE | 2.688 | 0.071801 |
| 11,12-DiHETrE | 19.459 | 4.11E-08 |
| 20-HETE | 15.722 | 7.76E-07 |
| 5(6)-EET | 0.663 | 0.517079 |
| 5,15-diHETE | 0.727 | 0.485537 |
| 5,6-DiHETE | 1.827 | 0.164976 |
| 5,6-DiHETrE | 0.423 | 0.656216 |
| 8(9)-EET | 0.435 | 0.648462 |
| 8-HETE | 0.337 | 0.714339 |
| LTB_4_ | 14.696 | 1.78E-06 |
| DHA | 1.323 | 0.269932 |
| 17-HDHA | 2.043 | 0.133857 |
| 14-HDHA | 2.243 | 0.110316 |
| 7-HDHA | 3.074 | 0.049617 |
| 4-HDHA | 1.054 | 0.351604 |
| 13-HDHA | 3.625 | 0.029389 |
| 8-HDHA | 2.005 | 0.138823 |
| Mar1 | 0.541 | 0.583201 |
| 16(17)-EpDPE | 0.230 | 0.794813 |
| 18-HEPE | 5.122 | 0.007233 |
| 15-HEPE | 2.720 | 0.069667 |
| 12-HEPE | 1.016 | 0.364982 |
| 5-HEPE | 0.660 | 0.51844 |
| 11-HEPE | 3.107 | 0.048091 |
| 18-HETE | 0.939 | 0.393756 |
| 19.20-DiHDPA | 16.139 | 5.55E-07 |
| 16-HETE | 1.233 | 0.294779 |
| 15-keto-PGE_2_ | 1.493 | 0.228475 |
| PGA_2_ | 7.083 | 0.001206 |
| PGB_2_ | 0.705 | 0.495925 |
| 6-keto-PGF_1a_ | 2.882 | 0.059665 |
| 2,3-dinor-11B-PGF_2a_ | 1.383 | 0.254483 |
| 5-iPF2a-VI | 0.023 | 0.977133 |
| 9-HETE | 1.081 | 0.342271 |
| 12-HHTrE | 8.920 | 0.000235 |
| 13-HOTrE | 1.917 | 0.151183 |
| 20-HDHA | 1.699 | 0.186952 |
| 11-HETE | 3.397 | 0.036482 |
| 16-HDHA | 2.969 | 0.054865 |
| 11-HDHA | 3.233 | 0.042647 |
| 19,20-EPDPE | 3.736 | 0.026466 |
| TXB_3_ | 7.558 | 0.000787 |
| 13,14-dihydro-15-keto PGF_2a_ | 3.189 | 0.044495 |
| 12,13-EpOME | 8.519 | 0.000335 |
| 17-HETE | 8.276 | 0.000415 |
| 14,15-DiHETrE | 25.082 | 6.29E-10 |
| 8,9-DiHETrE | 8.309 | 0.000403 |
| PGJ_2_ | 6.877 | 0.001453 |
| 15-Oxo ETE | 0.516 | 0.59801 |
| 14(15)-EET | 1.590 | 0.207953 |

**Supplementary Table 5.** Correlation between hematological variables and LM levels.

| **Lipid mediator*** |  | **Hematological variable** | |
| --- | --- | --- | --- |
|  | **Normality*** | **HGB** | |
|  | **P value** | **r** | **P value** |
| 12,13-EpOME | 5.71E-14 | 0.201 | 0.028 |
| 12,13-DiHOME | 8.70E-11 | 0.187 | 0.041 |
| 11,12-diHETrE | 2.00E-06 | 0.182 | 0.047 |
|  |  | **LYM count** | |
|  |  | **r** | **P value** |
| TXB_2_ | 3.28E-14 | 0.487 | 1.99E-08 |
| 12-HHTrE | 4.84E-15 | 0.481 | 3.09E-08 |
| PGJ_2_ | 2.35E-16 | 0.450 | 2.74E-07 |
| PGA_2_ | 2.88E-16 | 0.435 | 7.43E-07 |
| PGD_2_ | 3.30E-15 | 0.391 | 1.07E-05 |
| TXB_3_ | 8.75E-15 | 0.361 | 5.37E-05 |
| 11-HETE | 3.15E-15 | 0.333 | 2.18E-04 |
| PGF_2a_ | 4.38E-20 | 0.327 | 2.83E-04 |
| 5,15-diHETE | 9.47E-11 | 0.250 | 6.06E-03 |
| 11-HEPE | 1.02E-11 | 0.189 | 3.94E-02 |
| 19.20-DiHDPA | 2.55E-07 | -0.191 | 3.72E-02 |
| 17-HETE | 1.46E-06 | -0.216 | 1.83E-02 |
| 8,9-DIHETErE | 2.36E-04 | -0.228 | 1.26E-02 |
| 12,13-EpOME | 5.71E-14 | -0.278 | 2.21E-03 |
| LA | 1.16E-13 | -0.331 | 2.34E-04 |
| 11,12-diHETrE | 2.00E-06 | -0.360 | 5.69E-05 |
| 20-HETE | 8.64E-14 | -0.385 | 1.51E-05 |
| 14,15-DIHETErE | 1.33E-06 | -0.413 | 3.04E-06 |
|  |  | **NEUT count** | |
|  |  | **r** | **P value** |
| LTB_4_ | 1.90E-14 | 0.412 | 3.18E-06 |
| 19.20-DiHDPA | 2.55E-07 | 0.388 | 1.30E-05 |
| 14,15-DiHETErE | 1.33E-06 | 0.386 | 1.45E-05 |
| 20-HETE | 8.64E-14 | 0.369 | 3.60E-05 |
| 11,12-DiHETrE | 2.00E-06 | 0.351 | 9.05E-05 |
| 18-HEPE | 4.39E-13 | 0.305 | 7.38E-04 |
| 19,20-EPDPE | 8.70E-11 | 0.286 | 1.64E-03 |
| 17-HDHA | 9.37E-13 | 0.276 | 2.36E-03 |
| 12,13-EpOME | 5.71E-14 | 0.265 | 3.55E-03 |
| 9,10-diHOME | 4.00E-17 | 0.262 | 4.01E-03 |
| 15-HEPE | 7.01E-12 | 0.240 | 8.62E-03 |
| 5-HETE | 1.54E-09 | 0.226 | 1.34E-02 |
| LA | 1.16E-13 | 0.219 | 1.67E-02 |
| 17-HETE | 1.46E-06 | 0.203 | 2.68E-02 |
| 5,6-DiHETE | 3.40E-11 | 0.184 | 4.57E-02 |
| 20-HDHA | 9.36E-16 | 0.181 | 4.86E-02 |
| 9-HODE | 4.20E-09 | 0.180 | 4.97E-02 |
| 15-Oxo ETE | 3.08E-09 | -0.189 | 3.99E-02 |
| PGJ_2_ | 2.35E-16 | -0.189 | 3.99E-02 |
| PGF_2a_ | 4.38E-20 | -0.201 | 2.86E-02 |
| PGA_2_ | 2.88E-16 | -0.206 | 2.46E-02 |
| 12-HHTrE | 4.84E-15 | -0.227 | 1.31E-02 |
| TXB_3_ | 8.75E-15 | -0.239 | 8.90E-03 |
| TXB_2_ | 3.28E-14 | -0.265 | 3.63E-03 |
|  |  | **PLT count** | |
|  |  | **r** | **P value** |
| PGJ_2_ | 2.35E-16 | 0.330 | 2.47E-04 |
| PGD_2_ | 3.30E-15 | 0.321 | 3.78E-04 |
| PGA_2_ | 2.88E-16 | 0.316 | 4.56E-04 |
| TXB_2_ | 3.28E-14 | 0.303 | 8.00E-04 |
| 12-HHTrE | 4.84E-15 | 0.294 | 1.17E-03 |
| TXB_3_ | 8.75E-15 | 0.271 | 2.87E-03 |
| 5,15-diHETE | 9.47E-11 | 0.257 | 4.77E-03 |
| LTB_4_ | 1.90E-14 | 0.244 | 7.48E-03 |
| PGF_2a_ | 4.38E-20 | 0.242 | 7.95E-03 |
| 11-HETE | 3.15E-15 | 0.231 | 1.14E-02 |
| 20-HETE | 8.64E-14 | -0.181 | 4.92E-02 |
| 14,15-DIHETErE | 1.33E-06 | -0.182 | 4.80E-02 |
| 12,13-EpOME | 5.71E-14 | -0.219 | 1.67E-02 |
|  |  | **WBC count** | |
|  |  | **r** | **P value** |
| LTB_4_ | 1.90E-14 | 0.395 | 8.65E-06 |
| 18-HEPE | 4.39E-13 | 0.301 | 8.64E-04 |
| 20-HDHA | 9.36E-16 | 0.259 | 4.52E-03 |
| 16-HDHA | 1.99E-13 | 0.248 | 6.52E-03 |
| 19,20-EPDPE | 8.70E-11 | 0.243 | 7.76E-03 |
| 19.20-DiHDPA | 2.55E-07 | 0.220 | 1.64E-02 |
| 15-HEPE | 7.01E-12 | 0.216 | 1.82E-02 |
| 17-HDHA | 9.37E-13 | 0.192 | 3.67E-02 |
| Mar1 | 1.99E-13 | 0.248 | 6.52E-03 |

*The normality of hematological variables and each LMs concentration were examined by Shapiro-Wilk normality test. When the data followed a normal distribution, Pearson correlation was applied for the correlation analysis, otherwise Spearman correlation would be used. Only the LMs with the P value <0.05 are listed. HGB, hemoglobin; LYM, lymphocyte; NEUT, neutrophil; PLT, platelet; WBC, white blood cell.

**Supplementary Table 6**. Real-time RT-PCR primers and probes for H1N1 virus designed and used in this study.

| **Primer & Probe sets** | **Sequences** | **Gene target** | **Location (bp)** | **GenBank accession no.** |
| --- | --- | --- | --- | --- |
| H1N1 forward | GGAAAGAAATGCTGGATCTGGTA | Hemagglutinin | 822–844 | KP019929.1 |
| H1N1 reverse | ATGGGAGGCTGGTGTTTATAGC | Hemagglutinin | 904–925 |  |
| H1N1 probe | TAMRA-TGCAATACAACTTGTCARACACCCGAAGG-BHQ2 | Hemagglutinin | 874–902 |  |

**Supplementary Table 7**. Precursor ion, product ion, retention time, and the select internal standard for each lipid mediator.

| **Lipid mediator** | **Precursor** | **Product** | **RT (min)** | **Select IS** |
| --- | --- | --- | --- | --- |
| Lipoxin A_4_ | 351.3 | 115.1 | 3.2 | d4-PGE2 |
| Lipoxin A_4_ | 351.3 | 135.1 | 3.2 | d4-PGE2 |
| Lipoxin B_4_ | 351.3 | 129.2 | 2.72 | d4-PGE2 |
| Lipoxin B_4_ | 351.3 | 221 | 2.72 | d4-PGE2 |
| PGD_2_ | 351.2 | 189 | 2.81 | d4-PGE2 |
| PGD_2_ | 351.2 | 233 | 2.81 | d4-PGE2 |
| PGE_2_ | 351.2 | 271 | 2.53 | d4-PGE2 |
| PGE_2_ | 351.2 | 315 | 2.53 | d4-PGE2 |
| PGF_2a_ | 353.2 | 193 | 2.29 | d4-PGE2 |
| PGF_2a_ | 353.2 | 235 | 2.29 | d4-PGE2 |
| TXB_2_ | 369.2 | 169 | 2.2 | d4-PGE2 |
| TXB_2_ | 369.2 | 195.1 | 2.2 | d4-PGE2 |
| RvD1 | 375.2 | 141.1 | 3.27 | d4-PGE2 |
| RvD1 | 375.2 | 215.1 | 3.27 | d4-PGE2 |
| RvD2 | 375.3 | 175 | 2.86 | d4-PGE2 |
| RvD2 | 375.3 | 277 | 2.86 | d4-PGE2 |
| RvE1 | 349.3 | 129 | 1.55 | d4-PGE2 |
| RvE1 | 349.3 | 194.9 | 1.55 | d4-PGE2 |
| LA | 279.3 | 261.1 | 9.56 | d8-5s-HETE |
| 9,10-diHOME | 313.3 | 201 | 5.58 | d4-9,10-diHOME |
| 9,10-diHOME | 313.3 | 171.1 | 5.58 | d4-9,10-diHOME |
| 12,13-DiHOME | 313.3 | 183.1 | 5.38 | d4-9,10-diHOME |
| 12,13-DiHOME | 313.3 | 129.1 | 5.38 | d4-9,10-diHOME |
| 9-HODE | 295.1 | 171 | 7.05 | d4-9-HODE |
| 13-HODE | 295.2 | 195.2 | 7.01 | d4-LTB4 |
| 9(10)-HODE | 295.2 | 171.1 | 7.99 | d8-5s-HETE |
| ARA | 303.3 | 205.3 | 9.45 | d4-LTB4 |
| ARA | 303.3 | 259.3 | 9.45 | d4-LTB4 |
| 5-HETE | 319.3 | 115.1 | 7.61 | d4-PGE2 |
| 5-HETE | 319.3 | 203.3 | 761 | d4-PGE2 |
| 12-HETE | 319.3 | 179 | 7.47 | d4-PGE2 |
| 12-HETE | 319.3 | 135.1 | 7.47 | d4-PGE2 |
| 15-HETE | 319.3 | 175.1 | 7.21 | d4-LTB4 |
| 14(15)_EET | 319.2 | 175 | 7.96 | d4-LTB4 |
| 11(12)-DiHOME | 319.2 | 167 | 8.15 | d4-PGE2 |
| 11,12-diHETrE | 337.2 | 167 | 6.11 | d4-LTB4 |
| 11,12-diHETrE | 337.2 | 169.1 | 6.11 | d4-LTB4 |
| 20-HETE | 319.2 | 245 | 6.62 | d4-PGE2 |
| 5(6)-EET | 319.2 | 137 | 8.3 | d4-LTB4 |
| 5(6)-EET | 319.2 | 191 | 8.3 | d4-LTB4 |
| 5,15-DiHETE | 335.2 | 115 | 4.9 | d4-LTB4 |
| 5,15-DiHETE | 335.2 | 173.1 | 4.9 | d4-LTB4 |
| 5,6-DiHETE | 335.3 | 145.1 | 5.78 | d4-LTB4 |
| 5,6-DiHETE | 335.3 | 189.3 | 5.78 | d4-LTB4 |
| 5,6-DiHETrE | 337.2 | 145.1 | 6.57 | d4-LTB4 |
| 5,6-DiHETrE | 337.2 | 191.2 | 6.57 | d4-LTB4 |
| 8(9)-EET | 319.2 | 179.1 | 8.2 | d4-LTB4 |
| 8(9)-EET | 319.2 | 151.2 | 8.2 | d4-LTB4 |
| 8,9-DiHETrE | 337.2 | 127.1 | 6.27 | d4-LTB4 |
| 8,9-DiHETrE | 337.2 | 185.1 | 6.27 | d4-LTB4 |
| 8-HETE | 319.3 | 155.1 | 7.44 | d8-5s-HETE |
| 8-HETE | 319.3 | 163.1 | 7.44 | d8-5s-HETE |
| LTB4 | 335.2 | 195.1 | 5.1 | d4-LTB4 |
| LTB4 | 335.2 | 129.2 | 5.1 | d4-LTB4 |
| DHA | 327.4 | 229 | 9.31 | d4-PGE2 |
| DHA | 327.4 | 283 | 9.31 | d4-PGE2 |
| 17-HDHA | 343.2 | 201.1 | 7.32 | d4-LTB4 |
| 17-HDHA | 343.2 | 245 | 7.32 | d4-LTB4 |
| 14-HDHA | 343.2 | 205 | 7.37 | d8-5s-HETE |
| 14-HDHA | 343.2 | 234 | 7.37 | d8-5s-HETE |
| 7-HDHA | 343.3 | 141 | 7.49 | d4-PGE2 |
| 7-HDHA | 343.3 | 113.1 | 7.49 | d4-PGE2 |
| 4-HDHA | 343.3 | 101.1 | 7.76 | d4-PGE2 |
| 4-HDHA | 343.3 | 115 | 7.76 | d4-PGE2 |
| 10-HDHA | 343.3 | 153 | 7.38 | d4-PGE2 |
| 10-HDHA | 343.3 | 180.9 | 7.38 | d4-PGE2 |
| 13-HDHA | 343.3 | 192.9 | 7.31 | d4-PGE2 |
| 13-HDHA | 343.3 | 220.8 | 7.31 | d4-PGE2 |
| 8-HDHA | 343.3 | 109.1 | 7.55 | d8-5s-HETE |
| 8-HDHA | 343.3 | 189 | 7.55 | d8-5s-HETE |
| Mar1 | 359.3 | 113 | 5 | d4-PGE2 |
| Mar1 | 359.3 | 250 | 5 | d4-PGE2 |
| PD1 | 359.3 | 153.1 | 4.79 | d4-PGE2 |
| PD1 | 359.3 | 206.2 | 4.79 | d4-PGE2 |
| 16(17)-EpDPE | 343.3 | 233.1 | 8.02 | d4-PGE2 |
| 16(17)-EpDPE | 343.3 | 274.21 | 8.02 | d4-PGE2 |
| EPA | 301.2 | 177.1 | 8.94 | d8-5s-HETE |
| EPA | 301.2 | 257 | 8.94 | d8-5s-HETE |
| 18-HEPE | 317.2 | 215 | 6.44 | d4-PGE2 |
| 18-HEPE | 317.2 | 259 | 6.44 | d4-PGE2 |
| 15-HEPE | 317.2 | 175.1 | 6.69 | d8-5s-HETE |
| 15-HEPE | 317.2 | 219 | 6.69 | d8-5s-HETE |
| 12-HEPE | 317.2 | 179 | 6.84 | d4-9,10-diHOME |
| 12-HEPE | 317.2 | 208.1 | 6.84 | d4-9,10-diHOME |
| 5-HEPE | 317.2 | 115 | 6.94 | d8-5s-HETE |
| 5-HEPE | 317.2 | 201.1 | 6.94 | d8-5s-HETE |
| 11-HEPE | 317.2 | 167 | 6.73 | d8-5s-HETE |
| 11-HEPE | 317.2 | 195 | 6.73 | d8-5s-HETE |
| 8-HEPE | 317.2 | 127.1 | 6.79 | d4-LTB4 |
| 8-HEPE | 317.2 | 154.9 | 6.79 | d4-LTB4 |
| 10,17s-DiHDHA | 359.2 | 152.9 | 5.16 | d8-5s-HETE |
| 10,17s-DiHDHA-2 | 359.2 | 92.9 | 5.16 | d8-5s-HETE |
| 18-HETE | 319.2 | 261.3 | 7 | d8-5s-HETE |
| 19.20-DiHDPA | 361.2 | 273.2 | 6 | d4-LTB4 |
| 19.20-DiHDPA-2 | 361.2 | 87 | 6 | d4-LTB4 |
| 16-HETE | 319.2 | 189.2 | 7 | d8-5s-HETE |
| 16-HETE-2 | 319.2 | 233.3 | 7 | d8-5s-HETE |
| 15-keto-PGE_2_ | 349.2 | 113.1 | 3 | d4-PGE2 |
| 15-keto-PGE_2_-2 | 349.2 | 235.1 | 3 | d4-PGE2 |
| PGA_2_ | 333.2 | 189 | 4.1 | d4-PGE2 |
| PGB_2_ | 333.2 | 113.1 | 4.3 | d4-PGE2 |
| PGD_3_ | 349.2 | 189.1 | 2.3 | d4-PGE2 |
| PGD_3_-2 | 349.2 | 269.2 | 2.3 | d4-PGE2 |
| 13,14-dihydro-15-keto PGE_2_ | 351.1 | 175.1 | 3.4 | d4-PGE2 |
| 6-keto-PGF_1a_ | 369.2 | 163.1 | 1.5 | d4-PGE2 |
| 6-keto-PGF_1a_-2 | 369.2 | 245.2 | 1.5 | d4-PGE2 |
| 11B-PGE_2_ | 351.2 | 189.1 | 2.8 | d4-PGE2 |
| 11B-PGE_2_-2 | 351.2 | 271.3 | 2.8 | d4-PGE2 |
| 2,3-dinor-11B-PGF_2a_ | 325.2 | 144.9 | 1.4 | d4-PGE2 |
| 2,3-dinor-11B-PGF_2a_-2 | 325.2 | 163 | 1.4 | d4-PGE2 |
| 15-keto-PGF_2a_ | 351.2 | 219.1 | 2.7 | d4-PGE2 |
| 15-keto-PGF_2a_-2 | 351.2 | 289.2 | 2.7 | d4-PGE2 |
| 5-iPF2a-VI | 353.2 | 115 | 2.3 | d4-PGE2 |
| 9-HETE | 319.2 | 151 | 7.5 | d4-PGE2 |
| 12-HHTrE | 279.2 | 179 | 6.1 | d4-PGE2 |
| 12-HHTrE-2 | 279.2 | 134.8 | 6.1 | d4-PGE2 |
| 13-HOTrE | 293.2 | 195.2 | 6.5 | d4-LTB4 |
| 13-HOTrE-2 | 293.2 | 224.1 | 6.5 | d4-LTB4 |
| 20-HDHA | 343.2 | 241.1 | 7.21 | d8-5s-HETE |
| 20-HDHA-2 | 343.2 | 281.2 | 7.21 | d8-5s-HETE |
| 11-HETE | 319.2 | 166.9 | 7.4 | d8-5s-HETE |
| 16-HDHA | 343.2 | 233.2 | 7.4 | d8-5s-HETE |
| 16-HDHA-2 | 343.2 | 189 | 7.4 | d8-5s-HETE |
| 11-HDHA | 343.2 | 121 | 7.54 | d8-5s-HETE |
| 11-HDHA-2 | 343.2 | 148.8 | 7.54 | d8-5s-HETE |
| 9-HEPE | 317.2 | 166.9 | 7 | d8-5s-HETE |
| 9-HEPE-2 | 317.2 | 149 | 7 | d8-5s-HETE |
| 19,20-EPDPE | 343.2 | 241.2 | 7.91 | d8-5s-HETE |
| 19,20-EPDEP-2 | 343.2 | 281.3 | 7.91 | d8-5s-HETE |
| tetranor-12s-HETE | 265.2 | 109 | 5.85 | d4-PGE2 |
| 15-deoxy-12,14-PGD_2_ | 333.2 | 203.1 | 5.2 | d4-LTB4 |
| TXB_3_ | 367.2 | 169 | 1.6 | d8-5s-HETE |
| TXB_3_-2 | 367.2 | 195 | 1.6 | d4-PGE2 |
| 13,14-dihydro-15-keto PGF_2a_ | 353.2 | 113 | 3.27 | d4-PGE2 |
| PGE_1_ | 353.2 | 235.1 | 2.8 | d4-PGE2 |
| 9,10-EPOME | 295.2 | 171 | 8 | d4-PGE2 |
| 12,13-EpOME | 295.2 | 195.1 | 7.6 | d4-PGE2 |
| 12,13-EpOME-2 | 295.2 | 277.1 | 7.96 | d4-PGE2 |
| 12-epi LTB_4_ | 335.2 | 195 | 5.22 | d4-LTB4 |
| 17-HETE | 319.2 | 247.2 | 6.81 | d4-PGE2 |
| 17-HETE-2 | 319.2 | 203.1 | 6.81 | d4-PGE2 |
| 14,15-DIHETErE | 337.2 | 207 | 5.89 | d4-LTB4 |
| 8,9-DiHETErE | 337.2 | 126.9 | 6.34 | d4-LTB4 |
| PGJ_2_ | 333.2 | 189.1 | 4.19 | d4-PGE2 |
| PGD_2_ | 353.2 | 273.4 | 2.84 | d4-PGE2 |
| 9-Oxo ODE | 293.2 | 185.1 | 7.5 | d4-PGE2 |
| 13-Oxo ODE | 293.2 | 113.1 | 7.36 | d4-PGE2 |
| 15-Oxo ETE | 317.2 | 113 | 7.49 | d4-PGE2 |
| 15-DEOXY-12,14-PGJ_2_ | 315.2 | 203 | 6.7 | d4-PGE2 |
| 15-dexoy-12,14-PGJ_2_ | 315.2 | 271.2 | 6.7 | d4-PGE2 |
| 8-iso-PGF_2a_ | 353.3 | 193.3 | 2.01 | d4-PGE2 |
| 8-iso-PGF_2a_-2 | 353.3 | 309.1 | 2.01 | d4-PGE2 |
| 8-iso-15-keto-PGE_2_ | 349.3 | 253.1 | 3.04 | d4-PGE2 |
| 8-iso-15-keto-PGE_2_-2 | 349.3 | 287.1 | 3.04 | d4-PGE2 |
| 8-iso-15-keto-PGE_2_-2 | 349.3 | 113 | 3.04 | d4-PGE2 |
| d8-5s-HETE | 327.3 | 116 | 7.62 | IS |
| d4-LTB_4_ | 339.2 | 197 | 5.13 | IS |
| d4-9-HODE | 299.3 | 172 | 7.07 | IS |
| d4-9,10-DiHOME | 317.2 | 203 | 5.59 | IS |
| d4-PGE_2_ | 355.3 | 193.2 | 2.55 | IS |

RT，retention time; IS, internal standard
